# Alcohol Use and Alcohol-Related Seizures in Patients With Epilepsy

**DOI:** 10.3389/fneur.2018.00401

**Published:** 2018-06-05

**Authors:** Michael Hamerle, Leyli Ghaeni, Alexander Kowski, Florian Weissinger, Martin Holtkamp

**Affiliations:** ^1^Department of Cardiology, University Hospital Regensburg, Regensburg, Germany; ^2^Department of Neurology, Epilepsy-Center Berlin-Brandenburg, Charité—Universitätsmedizin Berlin, Berlin, Germany

**Keywords:** alcohol-related seizures, alcohol-drinking behavior, epilepsy, generalized genetic epilepsy, alcohols

## Abstract

**Purpose:** This study aimed to assess alcohol consumption and the occurrence of alcohol-related seizures in patients with epilepsy within the last 12 months.

**Methods:** In an epilepsy outpatient clinic, a standardized questionnaire was used to collect data retrospectively from consecutive adult epilepsy patients who had been suffering from the disease for at least 1 year. Logistic regression analyses were performed to identify independent predictors.

**Results:** A total of 310 patients with epilepsy were included. Of these, 204 subjects (65.8%) consumed alcohol within the last 12 months. Independent predictors for alcohol use were antiepileptic drug monotherapy (OR 1.901) and physicians' advice that a light alcohol intake is harmless (OR 4.102). Seizure worsening related to alcohol consumption was reported by 37 of the 204 patients (18.1%) who had used alcohol. All 37 subjects had consumed large quantities of alcohol prior to the occurrence of alcohol-related seizures regardless of their usual alcohol-drinking behavior. The amount of alcohol intake prior to alcohol-related seizures was at least 7 standard drinks, which is equivalent to 1.4 L of beer or 0.7 L of wine. In 95% of cases, alcohol-related seizures occurred within 12 h after cessation of alcohol intake. Independent predictors for alcohol-related seizures were generalized genetic epilepsy (OR 5.792) and chronic heavier alcohol use (OR 8.955).

**Conclusions:** Two-thirds of interviewed subjects had consumed alcohol within the last 12 months. This finding may be an underestimate due to patients' self-reporting and recall error. In all cases, the occurrence of alcohol related-seizures was associated with timely consumption of considerably large amounts of alcohol. Thus, a responsible alcohol intake seems to be safe for most patients with epilepsy. However, subjects with epilepsy and especially those with generalized genetic epilepsy should be made aware of an increased risk for seizures related to heavy alcohol consumption. Factors accompanying acute heavy alcohol intake such as altered sleep architecture, impaired adherence to antiepileptic medication, and metabolic disturbances may further facilitate the occurrence of seizures.

## Introduction

Alcohol consumption may trigger seizures in patients with epilepsy. Yet, there is currently little knowledge on the alcohol-drinking behavior of epilepsy patients. In the 1940s, William G. Lennox comprehensively analyzed alcohol consumption and the occurrence of alcohol-related seizures in 1,254 subjects with epilepsy (1). However, only about 30% of patients used alcohol, thus excluding 70% from any analysis of potential alcohol-related effects on the disease. The occurrence of alcohol-related seizures was reported by 21.1% of subjects who had used alcohol, and was more often stated by patients with symptomatic than with idiopathic or cryptogenic epilepsy (as classified at that time). Apart from this, there is little research on the occurrence of alcohol-related seizures in patients with epilepsy. A double-blinded, randomized, interventional study on 52 subjects with epilepsy demonstrated that a social alcohol intake over a 4-month-period did not increase seizure frequencies (2). In another interventional study on 14 patients with epilepsy and 10 healthy controls, acute moderate alcohol consumption initially suppressed epileptiform EEG-activity. Later however, when alcohol blood levels had declined, epileptiform EEG-activity was increased. Seizures occurred in some of those subjects and a rebound phenomenon was discussed (3).

Human and animal data have shown that acute alcohol intake has a biphasic effect on the central nervous system (CNS). Initially, the inhibitory gamma-aminobutyric acid (GABA)-ergic effect of alcohol exerts CNS depressant and anticonvulsant properties (4, 5). In the post-alcohol state, however, when alcohol blood levels decline, neuronal excitability is increased which may facilitate the occurrence of seizures in patients with epilepsy (6, 7).

The use of alcohol is very common in western societies (8). In Germany, 89% of all adults had consumed alcohol within the last 12 months (9). This makes it necessary for neurologists and other physicians to advise patients with epilepsy adequately on how to handle alcohol consumption with their chronic disease. The relationship between alcohol and epileptic seizures is complex. Research has mainly focused on the prevalence and pathophysiology of acute symptomatic seizures in the context of the alcohol withdrawal syndrome in alcohol-dependent subjects (10–12) and on the risk to develop epilepsy due to regular alcohol consumption (13, 14). However, there are only a few studies that have examined the patterns of alcohol drinking in subjects with a known history of epilepsy, and these are limited by outdated results or small sample sizes. In particular, data on seizure worsening associated with alcohol consumption in patients with epilepsy are very sparse. Therefore, we aimed (a) to systematically analyze the alcohol-drinking behavior of patients with epilepsy and (b) to identify independent predictors for alcohol use and the occurrence of alcohol-related seizures.

## Materials and methods

### Data collection using a standardized questionnaire, interview situations, and interview techniques

Between October 2008 and April 2010, consecutive patients treated at the Epilepsy Outpatient Clinic, Department of Neurology, Charité—Universitätsmedizin Berlin were informed about the study and invited to participate. The data collection on alcohol use was part of a research project systematically gathering information on nicotine, alcohol, and illicit drug use in epilepsy patients within the last 12 months. The data was collected by a standardized questionnaire (see Supplementary Material). We have published data on epilepsy and illicit drug use earlier (15). Only subjects ≥18 years who had suffered from epilepsy for at least 1 year were included. Epilepsy types and seizures were classified according to the International League Against Epilepsy (16). A single unprovoked seizure was defined as epilepsy if specific EEG alterations or causal brain lesions identified by magnetic resonance imaging (MRI) indicated an increased and enduring risk for further epileptic seizures (17). Subjects were excluded from participation if they had experienced status epilepticus or acute symptomatic seizures exclusively, if they had a history of psychogenic non-epileptic seizures, or if cognitive deficits, mental retardation or German language barrier impeded adequate understanding and reply to the questions. Patients with legal representatives were also not enrolled.

Prior to the interview, each participant was educated on the scientific background and purpose of the study. We placed great importance on a relaxed and informal interview atmosphere, and each subject was thoroughly informed that all moral aspects regarding nicotine, alcohol, and illicit drug use were irrelevant and that all data would be made anonymous and remain confidential. Thereby, we attempted to increase subjects' receptivity to the questions and avoid patients answering the questions in a more socially acceptable way. In several test-interviews, patients were intimidated when being asked about nicotine, alcohol, and illicit drug intake in front of their companions. Therefore, all interviews were held in a separate study room where only the interviewer and the patient were present. To ensure a standard and informal interview situation all patients were interviewed by the same person (MiHa) who was not one of the treating physicians at the Epilepsy Outpatient Clinic.

Alcohol consumption usually represents a taboo in the doctor-patient relationship and questions on the smoking status are answered more easily. Therefore, subjects were first queried about nicotine consumption and only later asked to give details on alcohol use. Toward the end of the interview, patients were questioned on illicit drugs. Study subjects passed through the domains of the questionnaire with an increasing social stigma degree.

In the opening question on alcohol use, subjects were asked: “Do you have any experience with alcohol consumption?” For this question, patients were able to respond in their own words and did not have to choose a predetermined response option. The interviewer carefully noted the given information on the quantity and frequency of alcohol consumption in the opening question. Subjects who had consumed alcohol within the last 12 months stated details on alcohol intake in the opening question and later by specifying the quantity and frequency of their individual alcohol consumption. Using that approach, the reliability of patients' responses on alcohol use could be evaluated regarding consistency. Data were excluded, if the patients' responses were inconsistent, if subjects were too hesitant to answer the questions, or if patients had refused to give details in only one of the interview's topics, that is nicotine, alcohol drinking and illicit drug use.

**Figure 1:**
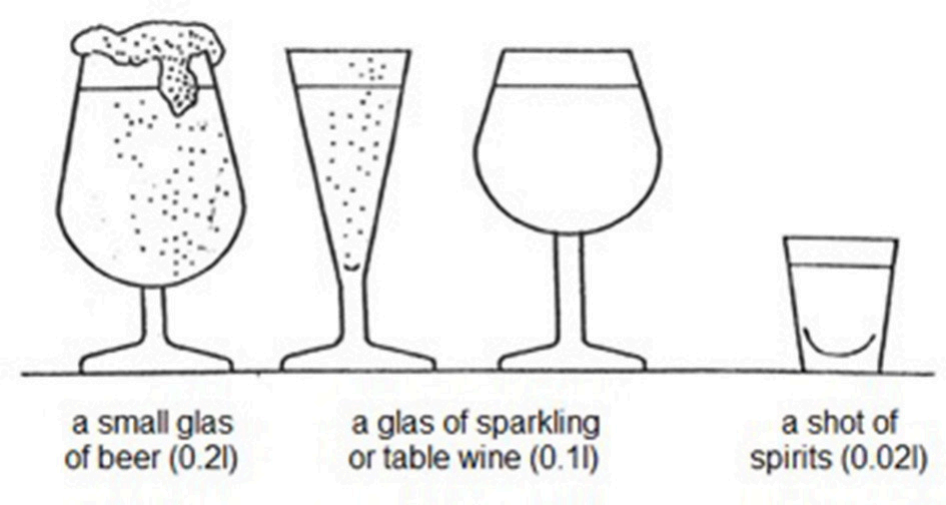
Amounts of different alcoholic beverages that correspond to 1 standard drink as defined by the World Health Organization. This illustration has been shown to the participants of this study to guide them in estimating their individual average alcohol intake per drinking occasion.

To ensure a comparable evaluation, alcohol consumption was translated and expressed in standard drinks containing 10 g of pure alcohol (18). To assist subjects in measuring their individual average alcohol intake per drinking occasion, a chart illustrating different alcoholic beverages containing a single standard drink was shown to each study participant (Figure 1). Regarding the usual frequency of alcohol consumption within the last 12 months, subjects were able to choose one out of the following different categories: daily, almost daily, 1–2 times a week, 1–2 times a month or < 1–2 times per month. According to that, patients who had consumed alcohol within the last 12 months were summarized in the following three alcohol drinking categories: Patients with alcohol intake of no more than 3–4 standard drinks daily, almost daily, 1–2 times per week or less than weekly were considered as light or occasional alcohol users. Moderate alcohol users were subjects who consumed more than occasional or light users but not more than 5–6 standard drinks daily, almost daily and not more than 9–10 standard drinks 1–2 times per week. Heavier alcohol use was considered as alcohol intake of more than 5–6 standard drinks daily, almost daily or more than 9–10 standard drinks 1–2 times per week. Alcohol abstinence was defined according to the World Health Organization (WHO) as a period of at least 12 months of non-consumption^1^.

The Alcohol Use Disorder Identification Test (AUDIT) is a 10-item core questionnaire developed by the WHO to identify hazardous and harmful alcohol intake (18) (Supplementary Material: questions 32–41), and was applied in all subjects who had consumed alcohol within the last 12 months. Patients are able to score up to a total of 40 points in domains like harmful alcohol intake and dependency symptoms. We considered patients as AUDIT-positive with AUDIT scores ≥8. This cut-off has been found to provide an accurate measure of harmful alcohol drinking across age, gender, and cultures (19).

Apart from that, all interviewed subjects were asked what their trusted neurologist or physician had told them regarding alcohol consumption in the context of their epilepsy. Patients were able to choose one out of four response options: (a) alcohol should be avoided completely, (b) alcohol can be consumed without any restriction, (c) light alcohol intake is harmless, or (d) no advice given by the physician.

### Alcohol-related seizures

In this study, an alcohol-related seizure was defined as a seizure in the context of epilepsy that occurred within short temporal relation to alcohol use (<24 h). Alcohol users were asked “Do you have experienced an alcohol-related seizure within the last 12 months?” If patients had experienced an alcohol-related seizure in the last 12 months, they were requested to recall details on the quantity of alcohol intake prior to the seizure and on the time between cessation of alcohol intake and seizure manifestation (<6 h/≥6–<12 h/≥12–<24h). The quantity of alcohol intake again was calculated and expressed in standard drinks to ensure a comparable evaluation (Figure 1). If patients had experienced more than one seizure related to alcohol use within the last 12 months, they were asked to state details on the seizure occurrence they remembered the best.

### Statistical analysis

Continuous data are presented as mean ± standard deviation (*SD*) or median where appropriate. Logistic regression analyses were used to calculate odds ratios with 95% confidence intervals as estimates for variables independently predicting alcohol use and the occurrence of alcohol-related seizures within the last 12 months.

In the logistic regression models, clinical data on patients' sex, age at interview, duration of epilepsy, epilepsy type, antiepileptic drug therapy, seizure frequency, alcohol drinking behavior over the last 12 months, and physicians' advice on alcohol use were included as possible confounding variables. In the results section of logistic regression analyses, findings were only noted if 95% CIs of the confounding variable did not include 1; if the 95% CI included 1, the corresponding variable was not significant and therefore was not pointed out. Statistical analyses were calculated using IBM SPSS statistics 24.0.

## Results

### Study population

The study population consisted of 310 patients with epilepsy (Table 1). Of these, seven subjects had suffered from only one single unprovoked seizure: In four of these patients remote structural brain lesions were demonstrated by neuroimaging indicating focal epilepsy. In one patient, interictal EEG findings were consistent with generalized genetic epilepsy, and in two subjects, EEG showed regional spikes and sharp waves without MRI structural brain lesions indicating focal epilepsy of unknown origin.

**Table 1:** Characteristics of the study population (*n* = 310).

**Variable**		**No. (%)/mean ±*SD*^a^**
Sex	Female	171 (55.2)
	Male	139 (44.8)
Age (in years)		44.7 ± 16.2
Duration of epilepsy (in years)		20.1 ± 16.8
Epilepsy type	Focal	213 (68.7)
	GGE^b^	67 (21.6)
	Unknown	30 (9.7)
AED^c^	Monotherapy	184 (59.4)
	Polytherapy	121 (39.0)
	No treatment	5 (1.6)
Seizure frequency	≥1/month	130 (41.9)
	<1/month	180 (58.1)
Alcohol use in the last 12 months	Alcohol abstinence	106 (34.2)
	Occasional or light use	147 (47.4)
	Moderate use	43 (13.9)
	Heavier use	14 (4.5)
Physicians' advice on the use of alcohol	Alcohol should be avoided completely	127 (41.0)
	No advice given	94 (30.3)
	Alcohol can be consumed without restriction	2 (0.6)
	Light alcohol intake is harmless	87 (28.1)

a *SD, standard deviation*.

b *GGE, generalized genetic epilepsy*.

c *AED, antiepileptic drug*.

### Alcohol consumption

Out of 310 interviewed subjects, 204 (65.8%) had used alcohol within the last 12 months, 158 (51%) within the last 30 days, and 108 (34.8%) within the last 7 days. Antiepileptic drug monotherapy (OR 1.901) and physicians' advice that a light alcohol intake is harmless (OR 4.102) were independent predictors for alcohol use within the last 12 months (Tables 2, 3). Out of the 204 patients who used alcohol, 147 (72%) were occasional or light alcohol users, 43 (21.1%) were moderate users and 14 subjects (6.9%) practiced heavier alcohol use. Nine subjects of the study population (2.9%) were AUDIT positive indicating hazardous and harmful alcohol use. All AUDIT positive subjects were heavier alcohol users.

**Table 2:** Possible confounding variables that were included in the logistic regression model regarding alcohol consumption within the last 12 months.

**Variable**		**Alcohol use within the last 12 months (*n* = 204)**	**Alcohol-abstinence (*n* = 106)**
		**No. (%)/mean ±*SD*^a^**	**No. (%)/mean ±*SD***
Sex	Female	108 (52.9)	63 (59.4)
	Male	96 (47.1)	43(40.6)
Age (in years)		43.8 ± 15.9	46.3 ± 16.7
Duration of epilepsy (in years)		18.9 ± 15.8	22.5 ± 18.4
Epilepsy type	Focal	135 (66.2)	78 (73.6)
	GGE^b^	45 (22.1)	22 (20.8)
	Unknown	24 (11.7)	6 (5.6)
AED^c^	Monotherapy	130 (63.7)	54 (50.9)
	Polytherapy	69 (33.8)	52 (49.1)
	No treatment	5 (2.5)	0
Seizure frequency	≥1/month	76 (37.3)	54 (50.9)
	<1/month	128 (62.7)	52 (49.1)
Physicians' advice	Alcohol should be avoided completely	73 (35.8)	54 (51)
	No advice	56 (27.4)	38 (35.8)
	Alcohol can be consumed without restriction	2 (1)	0
	Light alcohol intake is harmless	73 (35.8)	14 (13.2)

a *SD, standard deviation*.

b *GGE, generalized genetic epilepsy*.

c *AED, antiepileptic drug treatment*.

**Table 3:** Independent predictors for alcohol consumption within the last 12 months.

**Variable**		**OR^a^**	**95% CI^b^**	***P*-value**
AED^c^	Polytherapy	1.0 (ref.)		
	Monotherapy	1.901	1.152–3.138	*p* = 0.012
	None	N/A^d^	N/A	N/A
Physicians' advice	Alcohol should be avoided completely	1.0 (ref.)		
	Alcohol can be consumed without restriction	N/A	N/A	N/A
	No advice	1.043	0.599–1.814	*p* = 0.883
	Light alcohol intake is harmless	4.102	2.078–8.097	*p* <0.0001

a *OR, odds ratio*.

b *CI, confidence interval*.

c *AED, antiepileptic drug treatment*.

d *N/A, not available*.

Ninety-five patients (30.7%) were alcohol-experienced but had been abstinent in the last year. Eleven subjects 11 (3.5%) had never tried alcohol in their lifetime.

In alcohol-experienced subjects, who abstained from alcohol within the last 12 months (*n* = 95), epilepsy was reported to be the most common reason for no longer drinking alcohol (*n* = 50; 52.6%). Of those 50 patients, 49 subjects stated that they would consume alcohol if epilepsy had not been diagnosed and 16 patients stated that alcohol abstinence due to epilepsy is a challenge.

### Alcohol-related seizures

Thirty-seven out of 204 alcohol users (18.1%) had experienced alcohol-related seizures within the last 12 months (Table 4). In 95% (*n* = 35) of cases, these seizures had occurred within 12 h after cessation of alcohol intake.

**Table 4:** Clinical variables of patients with epilepsy who had experienced alcohol-related seizures within the last 12 months (*n* = 37).

**Patient-ID**	**Alcohol intake prior to alcohol-related seizures (standard drinks)**	**Time between cessation of alcohol intake and seizure occurrence (range in hours)**	**Usual alcohol-drinking behavior within the last 12 months**	**Epilepsy type**
#17	15	≥12– <24	Occasional or light use	Unknown
#25	8.75	<6	Occasional or light use	GGE ^a^
#31	15	≥6– <12	Occasional or light use	Focal
#32	10	≥6– <12	Occasional or light use	Focal
#38	34	<6	Heavier use	Focal
#40	7	≥6– <12	Moderate use	GGE
#53	12	<6	Occasional or light use	Focal
#54	17.5	<6	Occasional or light use	Focal
#63	15	≥6– <12	Occasional or light use	GGE
#65	12.5	≥6– <12	Moderate use	GGE
#77	7.5	<6	Occasional or light use	GGE
#81	20	<6	Heavier use	GGE
#83	7.5	≥6– <12	Heavier use	GGE
#87	12	<6	Moderate use	GGE
#116	7.5	<6	Occasional or light use	Focal
#133	Not remembered	<6	Heavier use	Focal
#140	12.5	<6	Moderate use	GGE
#141	Not remembered	≥6– <12	Heavier use	Focal
#144	12.5	≥6– <12	Occasional or light use	Focal
#147	15	<6	Occasional or light use	Focal
#154	10	≥12– <24	Moderate use	Focal
#178	15	<6	Occasional or light use	Unknown
#185	15	<6	Heavier use	Unknown
#188	14.5	<6	Occasional or light use	Focal
#199	11.5	<6	Occasional or light use	Focal
#223	Not remembered	<6	Heavier use	GGE
#258	15	<6	Moderate use	GGE
#264	7.5	<6	Occasional or light use	GGE
#272	15	<6	Occasional or light use	Unknown
#274	30	<6	Occasional or light use	GGE
#276	10.5	≥6– <12	Occasional or light use	GGE
#278	12.5	<6	Occasional or light use	GGE
#280	13	≥6– <12	Occasional or light use	Focal
#282	7.5	≥6– <12	Occasional or light use	Unknown
#283	10	<6	Occasional or light use	GGE
#291	8.75	<6	Moderate use	GGE
#308	15	≥6– <12	Heavier use	Focal

a *GGE, generalized genetic epilepsy*.

In multivariate analysis, subjects with heavier alcohol use in the last 12 months were more likely to experience alcohol-related seizures (OR 8.955), whereas occasional or light and moderate alcohol use was not associated with increased risk for alcohol-related seizures (Tables 5, 6). However, most of the patients (78.4%) who reported alcohol-related seizures were occasional, light or moderate alcohol users who had changed their usual alcohol intake toward higher consumption on the drinking occasion prior to the seizures (Table 4). The amount of alcohol intake before the occurrence of alcohol-related seizures was very high in all of the cases with a mean of 13.3 ± 5.8 standard drinks (median 12.5, range 7–34), which is equivalent to 2.5 L of beer or 1.25 L of wine. The minimum was 7 standard drinks, equivalent to ~1.4 L of beer or 0.7 L of wine.

**Table 5:** Possible confounding variables that were included in the logistic regression model regarding the occurrence of alcohol-related seizures in patients with epilepsy within the last 12 months.

**Variable**		**Alcohol-related seizure occurrence within the last 12 months (*n* = 37)**	**No alcohol-related seizures within the last 12 months (*n* = 167)**
		**No. (%)/mean ±*SD*^a^**	**No. (%)/mean ±*SD***
Sex	Female	17 (45.9)	91 (54.5)
	Male	20 (54.1)	76 (45.5)
Age (in years)		40.7 ± 14.7	44.5 ± 16.1
Duration of epilepsy (in years)		20.3 ± 15.1	18.6 ± 16
Epilepsy type	Focal	15 (40.6)	120 (71.9)
	GGE^b^	17 (45.9)	28 (16.8)
	Unknown	5 (13.5)	19 (11.3)
AED^c^	Monotherapy	22 (59.5)	108 (64.7)
	Polytherapy	14 (37.8)	55 (32.9)
	No treatment	1 (2.7)	4 (2.4)
Seizure frequency	≥1/month	13 (35.1)	63 (37.7)
	<1/month	24 (64.9)	104 (62.3)
Alcohol use within the last 12 months	Occasional or light use	22 (59.5)	125 (74.8)
	Moderate use	7 (18.9)	36 (21.6)
	Heavier use	8 (21.6)	6 (3.6)

a *SD, standard deviation*.

b *GGE, generalized genetic epilepsy*.

c *AED, antiepileptic drug*.

**Table 6:** Independent predictors for the occurrence of alcohol-related seizures within the last 12 months in patients with epilepsy.

**Variable**		**OR^a^**	**95% CI^b^**	***P*-value**
Epilepsy type	Focal	1.0 (ref.)		
	GGE^c^	5.792	2.427–13.823	*p* <0.0001
	Unknown	2.185	0.664–7.189	*p* = 0.198
Alcohol use within the last 12 months	Occasional or light use	1.0 (ref.)		
	Moderate use	0.819	0.306–2.194	*p* = 0.691
	Heavier use	8.955	2.625–30.545	*p* <0.0001

a *OR, odds ratio*.

b *CI, confidence interval*.

c *GGE, generalized genetic epilepsy*.

Patients with generalized genetic epilepsy (OR 5.792) were more likely to experience alcohol-related seizures compared to patients with focal epilepsy (Tables 5, 6). In patients with focal epilepsy, the mean amount of alcohol intake prior to alcohol-related seizures was 14.4 ± 6.5 (median 13, range 7.5–34) standard drinks, and in subjects with generalized genetic epilepsy 12.3 ± 5.9 (median 11.3, range 7–30). No significant difference was detected (*p* = 0.366).

In female patients, the mean amount of alcohol intake before alcohol-related seizures was 10.9 ± 3.1 standard drinks (median 11.3, range 7–15), and in male subjects, 15.4 ± 6.8 (median 15, range 7.5–34; *p* = 0.02).

Fifteen out of 95 (15.8%) alcohol-experienced but now abstinent subjects had experienced alcohol-related seizures in the past. In that group, the mean amount of alcohol intake prior to the seizures was 10.9 standard drinks. All of these patients stated that they had stopped alcohol consumption because of the experience of alcohol-related seizures.

## Discussion

In this study, we aimed to systematically analyze alcohol drinking and the occurrence of alcohol-related seizures in 310 epilepsy patients. Even though alcohol use may trigger seizures, 65% of interviewed subjects had consumed alcohol within the last 12 months and every third patient had consumed alcohol within the last 7 days. Our results are in line with previous population-based study findings from Canada reporting a 12-month prevalence of alcohol use in patients with epilepsy of 57.6% (20). In our study, most subjects were occasional or light alcohol users. Regarding chronic heavy alcohol consumption, our cohort of patients had used alcohol far more responsibly than the general adult German population. Only 2.9% of our interviewed study subjects were AUDIT positive indicating hazardous and harmful alcohol intake. By contrast, data from the general adult German population showed that a proportion of 19.7% is AUDIT positive (9).

In multivariate analysis, alcohol consumption within the last 12 months was independently related to AED monotherapy. It is highly likely that subjects with well-controlled epilepsies on monotherapy are more likely to consume alcoholic beverages than those with difficult-to-treat variants. Physicians' advice that “a light alcohol intake is harmless” was identified as an additional predictor for alcohol use. Patients with epilepsy may feel unsure about alcohol consumption on chronic medication and therefore may be willing to follow physicians' advices more often.

Thirty-seven out of 204 epilepsy patients who had consumed alcohol remembered that they had experienced an alcohol-related seizure within the last 12 months. These seizures occurred in the timely context of acute heavy alcohol consumption. The occurrence of seizures in short temporal relation to alcohol consumption may not prove that these seizures were necessarily causally related to alcohol use. The following arguments however support this hypothesis: Most subjects with alcohol-related seizures were occasional, light or moderate alcohol users but a noticeable change in their usual alcohol-drinking behavior toward higher consumption prior to the seizures could be documented. This taken together with the fact that almost all alcohol-related seizures (95%) had occurred within the first 12 h after cessation of alcohol intake support a causal relationship between alcohol use and temporally close seizure manifestation in these cases.

In the study population, generalized genetic epilepsy was an independent predictor for the occurrence of alcohol-related seizures. The mean alcohol intake prior to alcohol-related seizures was not higher in patients with generalized genetic epilepsy than in subjects with focal epilepsy. Lennox stated that alcohol-related seizures had occurred more often in patients with symptomatic than in cryptogenic or idiopathic epilepsies (1). The then applied syndromatic allocation, however, may not be in exact conformance with the present classifications (16, 17). Janz (21) later observed that alcohol-related seizures were more likely to occur in subjects with generalized genetic epilepsy than in those with focal epilepsy, which is consistent with our findings (21).

Acute alcohol consumption suppresses central nervous excitability by activating the inhibitory GABA-system (22). GABA is the major inhibitory neurotransmitter in the brain. Furthermore, alcohol inhibits glutamate activity, which is the major excitatory neurotransmitter of the CNS. Thus in subjects with epilepsy, alcohol intake initially reduces CNS epileptiform EEG-activity. Later however, when alcohol blood levels decline, epileptiform EEG-activity has been shown to be increased which is associated with a higher risk for seizures (4–6, 23). In an experimental study on mice with chronic epilepsy, seizure thresholds were measured after the administration of ethanol. Initially, anticonvulsant properties of ethanol were observed, but later a transient lowering of seizure thresholds and hyper-susceptibility to seizures were reported (7).

In patients with generalized genetic epilepsy, seizures commonly manifest within 30 min after awakening. A transcranial magnetic stimulation study on patients with genetic generalized epilepsy demonstrated that motor cortex excitability was significantly increased in the early morning (24). In subjects with generalized genetic epilepsy, this increased neuronal excitability in the early morning may be potentiated by the hyper-excitable post-alcohol state, and this effect may be responsible for the increased susceptibility to alcohol-related seizures compared to focal epilepsy.

### Clinical perspective

Most of our interviewed subjects (>80%) that consumed alcohol within the last 12 months did not experience alcohol-related seizures. Current data on the quantity of alcohol intake prior to the occurrence of alcohol-related seizures in patients with epilepsy highly suggest that these situations are related to the acute consumption of considerably large amounts of alcohol. Subjects who reported alcohol-related seizures had consumed at least 7 standard drinks before seizures occurred which is equivalent to 1.4 L of beer or 0.7 L of wine. Occasional or light and moderate alcohol-drinking behavior was not associated with alcohol-related seizure occurrences. In the general German population, 89% of all adults had used alcohol within the last 12 months, only 8% were alcohol-experienced but abstinent, and 3% had never used alcohol in their lifetime (9). In the present study, 30.7% of patients were alcohol-experienced but abstinent and 3.5% had never consumed alcohol in their lifetime. Therefore, the proportion of alcohol-experienced but abstinent subjects with epilepsy was almost four times higher than in the general population. Epilepsy was often stated to be the only reason for alcohol-abstinence, which felt challenging to many subjects. Alcohol abstinence may not be necessary as long as epilepsy patients practice a responsible alcohol intake. Subjects with generalized genetic epilepsy however should be made aware of their increased susceptibility to alcohol-related seizures.

### Limitations

Our systematic data collection based on personal interviews allowed us to provide updated knowledge on the patterns of alcohol drinking and the occurrence of alcohol-related seizures in a large cohort of 310 epilepsy patients.

Several limitations have to be discussed. First, our data on alcohol use depended on patients' self-reporting and may be affected by recall bias. It has been demonstrated that assessing alcohol consumption is biased by recall even when the recall period is only 1 week (25). In our study population, alcohol consumption is probably underestimated. However, this does not impact our main findings. Moreover, patients were seen at our institution at scheduled outpatient visits and did not attend the clinic after acute manifestations of alcohol-related seizures. Only a minority of patients documented details on alcohol-related seizures in seizure diaries. Our retrospective data collection on alcohol-related seizures also depended on subjects' recall capability, and may reflect bias due to recall errors. We addressed this by focusing only on alcohol-related seizures that had occurred within the last 12 months. Details were only recorded on those alcohol-related seizures that subjects were able to remember the best. As a consequence however, alcohol-related seizures may have also occurred after smaller amounts of alcohol intake or in other circumstances that were not taken into account in the present study.

Second, as patients were interviewed retrospectively on the occurrence of alcohol-related seizures, we were not able to provide data on AED drug levels after the acute manifestation of these seizures. We cannot exclude that subjects might have been more prone to seizure occurrences due to AED non-adherence. Furthermore, we cannot exclude hypoglycemic episodes caused by acute heavy alcohol consumption (26), which may have contributed to the manifestation of epileptic seizures (27).

Third, other studies have shown that alcohol consumption and especially the consumption of considerable large amounts of alcohol may reduce sleep quality by increasing light sleep and wake-up periods during the second half of the night time sleep period (28, 29). In addition to that, alcohol intake significantly suppresses REM sleep periods (30). Reduced sleep quality and consecutive sleep deprivation have long been discussed to facilitate the occurrence of seizures in patients with epilepsy (31), and especially in those with generalized genetic epilepsy (32–34). Altered sleep architecture due to acute alcohol consumption constitutes a non-negligible and important co-factor for seizure risk in patients with epilepsy. Due to the retrospective design of the present study, we were not able to assess sleep quality prior to alcohol-related seizure occurrences. Future prospective research, e.g., using polysomnography, will be needed to provide insight into the complex relationship between alcohol consumption, altered sleep architecture and timely manifestation of seizures.

Finally, the present study population was exclusively recruited at a tertiary care epilepsy center where usually patients with more severe variants of the disease are treated. This indicates a potential selection bias and our results may not be generalized to all epilepsy patients without restrictions.

## Ethics statement

The study was approved by the local Institutional Review Board (EA 1/146/08), and signed informed consent was obtained from all participants.

## Author contributions

MiH: data collection, statistical, analysis, wrote manuscript. LG, AK, and FW helped recruiting patients, helped to improve manuscript. MaH: statistical analysis, wrote manuscript.

### Conflict of interest statement

The authors declare that the research was conducted in the absence of any commercial or financial relationships that could be construed as a potential conflict of interest.

## Footnotes

^1^*Lexicon of Alcohol and Drug Terms*. WHO. Available online at: http://www.who.int/substance_abuse/terminology/who_lexicon/en/
