# Supplementary material for: Alcohol Use and Alcohol-Related Seizures in Patients With Epilepsy
Source: Front Neurol. 2018 Jun 5;9:401. doi: 10.3389/fneur.2018.00401 (PMC5996121; doi:10.3389/fneur.2018.00401)
Supplement: Supplementary file 1 [file Data_Sheet_1.DOCX]

**Supplementary material**

The study questionnaire was originally in German language


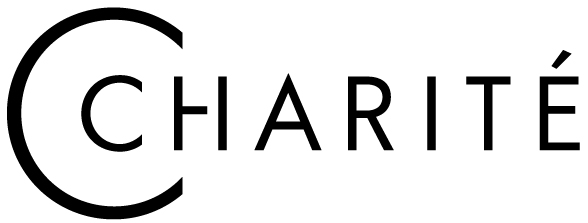


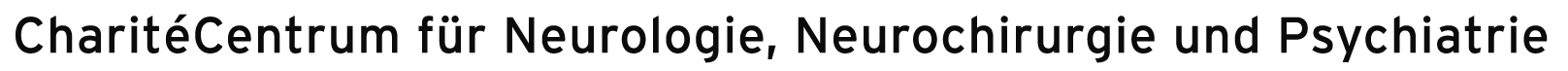


Charité ⏐ Campus Mitte ⏐ 10098 Berlin

**Nicotine, alcohol and illicit drug use in patients with epilepsy**

I) Demographic details and medical history

1. Patient-ID ­___________

2. Age (in years) ___________

3. Sex □ Male

□ Female

4. Duration of epilepsy (in years) ___________

5. Epilepsy type □ Focal

□ GGE

□ Unknown

6. Antiepileptic drug (AED) therapy □ Monotherapy

□ Polytherapy

□ No treatment

7. Seizure frequency □ ≥ 1/month

□ < 1/month

II) Epilepsy and nicotine consumption

8. Have you made any experiences with smoking/ nicotine consumption?

□ Current smoker (daily or almost daily within the last 12 months)

□ Occasional smoker (not daily or almost daily within the last 12 months/ ≥100 cigarettes)

□ Ex-Smoker , Pack-years: __________

□ Non-Smoker

9. Have you smoked daily or almost daily in the last 12 months?

□ Yes

□ No (►question 16)

10. How soon after you wake up do you smoke your first cigarette?

□ 5 minutes (3 points)

□ 6-30 minutes (2 points)

□ 31-60 minutes (1 point)

□ 60 minutes or more (0)

11. Do you find it difficult to refrain from smoking in places where it is forbidden (e.g. shopping mall, restaurant)?

□ Yes (1 point)

□ No (0)

12. Which cigarette would you hate most to give up?

□ The first one in the morning (1 point)

□ Any other (0)

13. Do you smoke more frequently during the first hours after waking up than the rest of the day?

□ Yes (1 point)

□ No (0)

14. Do you smoke if you are so ill that you are in bed most of the day?

□ Yes (1 point)

□ No (0)

15. How many cigarettes do you smoke every day?

□ 31 or more (3 points)

□ 21-30 (2 points)

□ 11-20 (1 point)

□ 10 or less (0)

16. Have you experienced a seizure in close temporal relation of nicotine consumption (within 30 min) within the last 12 months?

□ Yes

□ No

17. In what temporal relationship did the seizure occur after nicotine consumption?

□ While smoking

□ 1-15min after smoking

□ 16-30min after smoking

18. How much cigarettes did you consume before seizure occurrence?

___________ cigarettes

19. What did your trusted physician advise you regarding nicotine consumption?

□ No advice given by the physician

□ Cigarettes can be consumed without any restriction

□ Smoking cigarettes may worsen seizures

III) Epilepsy and alcohol consumption

20. Do you have any experience with alcohol consumption?

□ Yes

Interviewer notes

*(How often? How much? Preferred alcoholic beverage? When was the last drinking occasion? Reasons for abstinence?)*

________________________________________________________________________________________________________________________________________________________________________________________________________________________________________________________________________________________________________________________________________________________________________________________________________________________________________________________________________________________________________________________________________________________________________________________________________________________

□ No

21. Have you used alcohol within the last 12 months?

□ Yes (►question 28)

□ No (►question 24)

22. Have you used alcohol within the last 30 days?

□ Yes

□ No

23. Have you used alcohol within the last 7 days?

□ Yes

□ No

24. Can you give me reasons why you have not used alcohol within the last 12 months?

□ Epilepsy

□ Other reason(s) ___________________________________________

25. Have you consumed more alcohol before epilepsy was diagnosed?

□ Yes

□ No

26. Would you consume alcohol, if epilepsy had not been diagnosed?

□ Yes

□ No

27. Is alcohol-abstinence due to epilepsy a challenge in your lifestyle?

□ Yes

□ No

28. On what occasion(s) do you consume alcohol?

□ After physical activity/ sports

□ With dinner/ meals

□ Celebrations/ festivities/ ceremonies

□ For relaxation

□ While watching TV

□ Other occasions ____________________________________________________________

29. Which sort of alcoholic drink do you prefer?

□ Beer

□ Table wine or sparkling wine

□ Long-Drinks or Cocktails

□ Spirits/ hard liquor

□ Other drinks _______________________________________________________________

30. How often do you consume alcohol usually?

□ Daily

□ Almost daily

□ On the weekends only

□ 1-2 times per week

□ 1-2 times per month

□ 1-2 times per six months

□ 1-2 times per year

31. How much alcohol do you consume per usual drinking occasion?

Amount of alcohol intake ____________________

Standard drink calculation ____________________

AUDIT questionnaire

32. How often do you have a drink containing alcohol?

□ Never (0)

□ Monthly or less (1 point)

□ 2 to 4 times a month (2 points)

□ 2 to 3 times a week (3 points)

□ 4 or more times a week (4 points)

33. How many drinks containing alcohol do you have on a typical day when you are drinking?

□ 1 or 2 (0)

□ 3 or 4 (1 point)

□ 5 or 6 (2 points)

□ 7,8 or 9 (3 points)

□ 10 or more (4 points)

34. How often do you have six or more drinks on one occasion?

□ Never (0)

□ Less than monthly (1 point)

□ Monthly (2 points)

□ Weekly (3 points)

□ Daily or almost daily (4 points)

35. How often during the last year have you found that you were not able to stop drinking once you started?

□ Never (0)

□ Less than monthly (1 point)

□ Monthly (2 points)

□ Weekly (3 points)

□ Daily or almost daily (4 points)

36. How often during the last year have you failed to do what was normally expected from you because of drinking?

□ Never (0)

□ Less than monthly (1 point)

□ Monthly (2 points)

□ Weekly (3 points)

□ Daily or almost daily (4 points)

37. How often during the last year have you needed a first drink in the morning to get yourself going after a heavy drink session?

□ Never (0)

□ Less than monthly (1 point)

□ Monthly (2 points)

□ Weekly (3 points)

□ Daily or almost daily (4 points)

38. How often during the last year have you had a feeling of guilt or remorse after drinking?

□ Never (0)

□ Less than monthly (1 point)

□ Monthly (2 points)

□ Weekly (3 points)

□ Daily or almost daily (4 points)

39. How often during the last year have you been unable to remember what happened the night before because you had been drinking?

□ Never (0)

□ Less than monthly (1 point)

□ Monthly (2 points)

□ Weekly (3 points)

□ Daily or almost daily (4 points)

40. Have you or someone else been injured as a result of your drinking?

□ No (0)

□ Yes, but not in the last year (2 points)

□ Yes, during the last year (4 points)

41. Has a relative or friend or a doctor or another health worker been concerned about your drinking or suggested you cut down?

□ No (0)

□ Yes, but not in the last year (2 points)

□ Yes, during the last year (4 points)

Alcohol-related seizures

42. Have you experienced an alcohol-related seizure within the last 12 months?

□ Yes

□ No

43. In what temporal relation did the seizure occur after cessation of alcohol intake?

□ <6h

□ ≥ 6h and <12h

□ ≥ 12h and <24h

44. How much alcohol did you consume prior to the seizure occurrence?

Amount of alcohol intake ____________________

Standard drink calculation ____________________

45. What has your trusted physician told you regarding epilepsy and the use of alcohol?

□ Alcohol should be avoided completely

□ Alcohol can be consumed without any restriction

□ Light alcohol intake is harmless

□ No advice given by the physician

IV) Epilepsy and illicit drug use

46. Have you ever heard about cannabis use as an alternative add-on-therapy in the management of epilepsy?

□ Yes

□ No (►question 48)

47. What information on epilepsy and cannabis use have you received?

□ Cannabis use has no effect on the disease

□ Epilepsy patients may experience beneficial effects

□ Cannabis use may lead to seizure worsening in patients with epilepsy

48. What do YOU think about the effects of cannabis use on epilepsy?

□ Cannabis use has no effect on the disease

□ Epilepsy patients may experience beneficial effects

□ Cannabis use may lead to seizure worsening in patients with epilepsy

49. Have you made any experience with cannabis use on your own?

□ Yes, within the last 12 months

□ Yes, but not within the last 12 months (►question 53)

□ No (►question 60)

50. What form of cannabis consumption have you usually used within the last 12 months?

____________________________________________________________________________________________________________________________________________________________________________________________________________________________________________________________________________________________________________

51. How often have you consumed cannabis within the last 12 months?

□ Daily

□ Almost daily

□ At least once per week

□ At least once per month

□ Every other month

□ Other ____________________________________________________________________

□ Less than 10 times in the last 12 months

52. For what reasons have you consumed cannabis within the last 12 months? (►question 58)

□ Relaxation

□ Enjoyment

□ Parties/ celebrations / festivities

□ Curiosity

□ Other reasons ______________________________________________________________

53. Why have you stopped using cannabis?

□ Epilepsy

□ Other reasons ______________________________________________________________

54. When have you consumed cannabis the last time (date/year)?

___________________________________________________________________________

55. What forms of cannabis have you used in the past?

____________________________________________________________________________________________________________________________________________________________________________________________________________________________________________________________________________________________________________

56. How often have you used cannabis in the past?

□ Daily

□ Almost daily

□ At least once per week

□ At least once per month

□ Every other month

□ Other ______________________________________________________________

□ Less than 10 times in the past

57. For what reasons have you consumed cannabis in the past?

□ Relaxation

□ Enjoyment

□ Parties/ celebrations / festivities

□ Curiosity

□ Other reasons ______________________________________________________________

58. Have you consumed cannabis after epilepsy was diagnosed?

□ Yes

□ No

59. Have you experienced any effect of cannabis use on your epilepsy?

____________________________________________________________________________________________________________________________________________________________________________________________________________________________________________________________________________________________________________

60. Have you made any experience with illicit drug use (other than cannabis)?

□ Yes, within the last 12 months

□ Yes, but not within the last 12 months (►question 64)

□ No (►end of interview)

61. Which illicit drugs (other than cannabis) have you used within the last 12 months?

____________________________________________________________________________________________________________________________________________________________________________________________________________________________________________________________________________________________________________

62. How often have you used illicit drugs (other than cannabis) within the last 12 months? Please state details on each consumed drug respectively!

____________________________________________________________________________________________________________________________________________________________________________________________________________________________________________________________________________________________________________

63. For what reasons have you used illicit drugs (other than cannabis) within the last 12 months? (►question 69)

□ Relaxation

□ Enjoyment

□ Parties/ celebrations / festivities

□ Curiosity

□ Other reasons ______________________________________________________________

64. Why have you stopped using illicit drugs (other than cannabis)?

□ Epilepsy

□ Other reason __________________________________________________­­_____________

65. When have you consumed illicit drugs (other than cannabis) the last time (date/year)?

___________________________________________________________________________

66. Have you consumed illicit drugs (other than cannabis) after epilepsy was diagnosed?

□ Yes

□ No

67. Have you experienced any effect of illicit drug use (other than cannabis) on your epilepsy?

____________________________________________________________________________________________________________________________________________________________________________________________________________________________________________________________________________________________________________
